# Extra‐CNS metastasis of glioblastoma multiforme to cervical lymph nodes and parotid gland: A case report

**DOI:** 10.1002/ccr3.2985

**Published:** 2020-05-28

**Authors:** Sami Alhoulaiby, Ali Abdulrahman, Ghazal Alouni, Mufed Mahfoud, Zuhair Shihabi

**Affiliations:** ^1^ Eye Surgical Hospital Ministry of Health Damascus Syria; ^2^ Faculty of Medicine Tishreen University Latakia Syria; ^3^ Department of Neurosurgery Tishreen University hospital Latakia Syria; ^4^ Department of Pathology Tishreen University Latakia Syria

**Keywords:** extra‐CNS metastasis, glioblastoma, lymph node metastasis, neurosurgery, parotid gland metastasis

## Abstract

Extra CNS metastasis of glioblastoma multiforme is extremely rare. We report a case of a 53‐year‐old Caucasian male who, after undergoing surgical resection and nine months adjuvant therapy, had a recurrence of the cancer with an infiltration expanding outside the cranium to the left maxilla, mandible and parotid gland.

## Graphical abstract

The metastasis of brain tumors outside the central nervous system is extremely rare yet plausible and should be ruled out even after the complete regimen of treatment is given (surgery, radiation, and temozolomide) as it doesn't necessarily respond successfully leading to a poor prognosis.

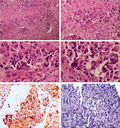

## 1 INTRODUCTION

Glioblastoma multiforme (GBM) is the most common and aggressive primary brain tumor in adults.^1^ It makes up 45% of all gliomas and 16% of primary brain tumors.^2^ The World Health Organization (WHO) classified this malignancy as a grade IV tumor in 2007 and then added it to the diffuse gliomas in the 2016‐update.^3^, ^4^ The incidence of GBM in Europe and North America is 2‐3 per 100000 adults/year with a slightly higher incidence in men than in women (1.26:1).^5^ It usually presents in late adulthood with a median age of 64 years, but can also occur at any age including childhood.^2^, ^6^ The current treatment method of GBM is based on surgical resection succeeded by concurrent temozolomide with radiation therapy and then followed by a maintenance dose of temozolomide.^7^

GBM rarely spreads outside the central nervous system (CNS), and metastases are estimated to only occur in 0.4%‐0.5% of all patients with GBM.^8^ We have found approximately 150 cases of extra‐CNS metastases in English literature up‐to‐date,^9^ the most common sites of GBM metastases were the lungs, pleura, lymph nodes, liver, bone, and neuroaxis. The seeding rarely takes place in the skin, parotid gland, and other organs as well. Awan et al thoroughly explored published cases on the topic in their review.^10^

The prognosis of GBM is poor. The median overall survival from initial diagnosis is 13 ± 2.4 months, whereas GBM with extra‐CNS metastases is associated with a more dismal prognosis and median overall survival from diagnosis of metastasis of 6 ± 0.8 months.^9^ Lun et al analyzed extensively the data regarding the prognosis of the disease with and without extra‐CNS metastasis, and the efficacy of treatment on the results.^8^

In this article, we present a case of a 53‐year‐old male patient who had a GBM that metastasized to the cervical lymph nodes and the left parotid gland.

## 2 CASE PRESENTATION

A 53‐year‐old Caucasian male presented to the neurology clinic at Tishreen University hospital in Latakia, Syria, complaining of a 3‐day occipitofrontal headache, forgetfulness, slurred speech, and weakness in the lower extremities. The headache was severe, gradually increasing, waking the patient up at night, and not responding to analgesics. Physical examination confirmed the lower extremity paraplegia, but found no other abnormalities: no vomiting, fever, vertigo, altered mental status, nor thoracic or abdominal pain. Laboratory tests were normal as well. The patient reported a 10‐year history of medically treated hypertension and herpetic encephalitis 4 months earlier. Family history is remarkable for cerebrovascular accidents (CVA) in his father and two of his sisters at a young age (younger than 60 years). The patient's medication at the time of admission included aspirin, bisoprolol, ramipril, amlodipine, valsartan, and hydrochlorothiazide for hypertension and cardiovascular protection, and carbamazepine following the herpetic encephalitic episode.

Based on the aforementioned clinical findings, the primary differential diagnosis included CVA and space‐occupying lesions (ie, tumor or abscess). Imaging workup followed, and results are as follows:

Computerized tomography (CT) scan: a low density irregularly bordered mass with foci of hemorrhage and surrounding edema in the left temporoparietal region. A 10 mm midline shift toward the right and compression of the left lateral ventricle.

Magnetic resonance imaging (MRI) scan: similar findings of falx cerebri deviation to the right with a collapse of the left lateral ventricle. This collapse was caused by a mass contrast‐enhancing lesion with irregular borders in the left temporoparietal region, enclosed with edema which was causing an extreme mass effect.

**FIGURE 1:**
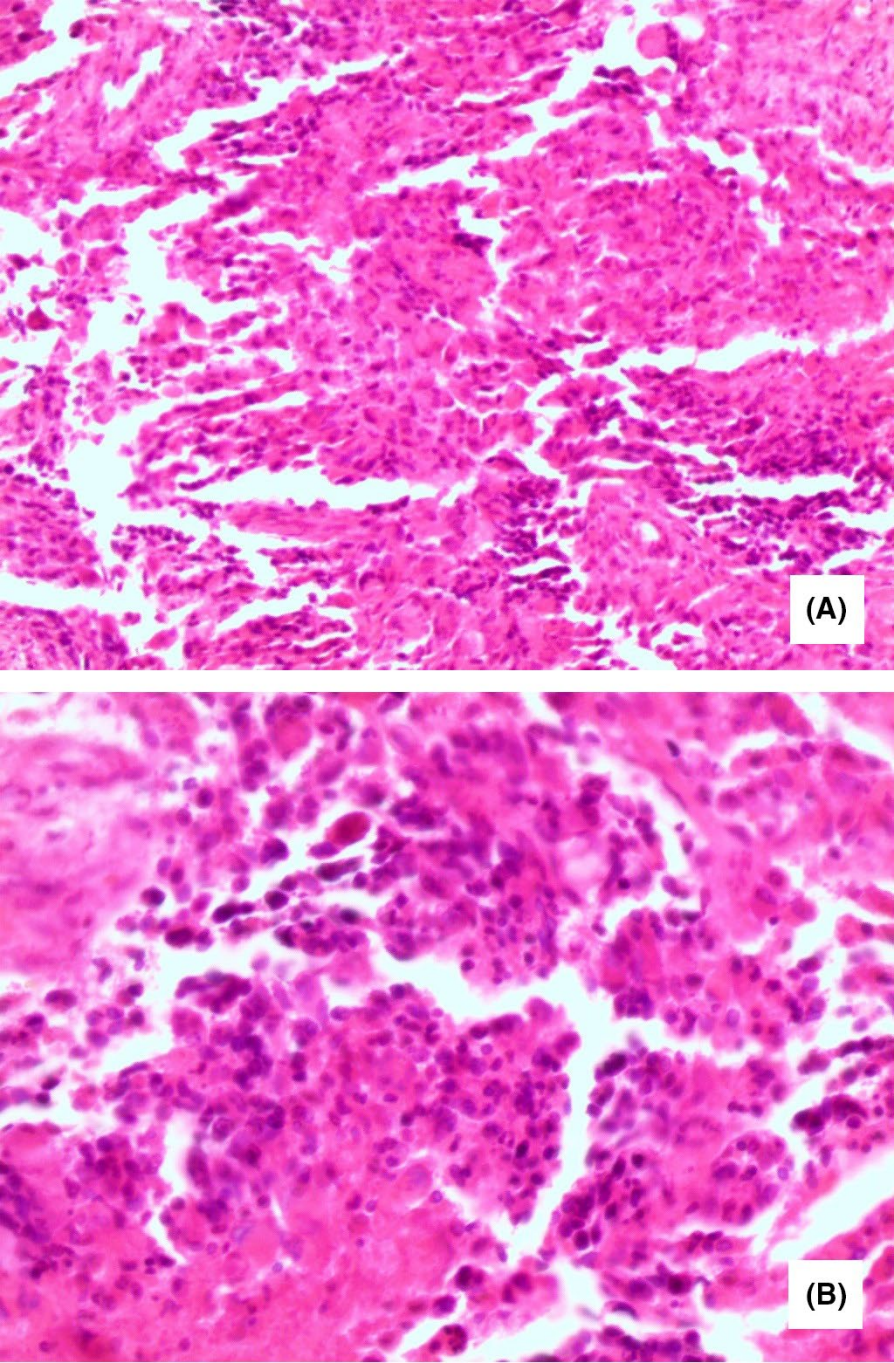
Pathology of the brain tumor showing: (A) Diffuse proliferation of variable sized anaplastic cells occurring in small groups and isolated forms with large round or elongated hyperchromatic nuclei, and occasional bizarre mitotic figures (H&E ×200). B, Higher magnification with scattered foci of necrosis (H&E ×400)

The patient underwent craniotomy and resection of the brain lesion. The specimen was sent to the pathology laboratory, which described it as follows: Macroscopically: multiple fragments of tissue that measured together 5 cm in their greatest dimension. Cut sections revealed a gray‐yellowish soft tissue with abundant necrosis and hemorrhage. Microscopically: a malignant cellular tumor of large, pleomorphic, hyperchromatic cells. Other features included numerous mitoses, microvascular proliferation, necrosis, and pseudo‐palisaded nuclei. Fibrillar background and focal astrocytic differentiation also accompanied the pathologic presentation. The pathologist identified the tumor as a WHO grade IV glioblastoma multiforme (GBM) (Figure 1).

After surgery, the patient at first had disturbed consciousness which resolved completely in the next few days. However, he developed signs of infection. White blood cell (WBC) level rose to 22 × 10^3^ cell/μL and CRP to 90 mg/L. He was treated with a 10‐day regimen of intravenous (IV) ceftriaxone‐sulbactam [1 g, twice daily] and IV vancomycin [1 g, twice daily]. The patient was also given mannitol for cerebral edema [50 mL/every 2 hours reduced later to 25 mL/every 2 hours] along with enoxaparin and dexamethasone [8 mg, three times a day].

After inpatient stability and discharge, adjuvant therapy was initiated with 2 months of radiation therapy to the clinical target volume (CTV). The total radiation dose was 60 Gray (Gy) divided on 2 Gy per session, five times a week. Directly after the radiation sessions ended, a 6‐month temozolomide treatment was ensued. The chemotherapy was administered with 250 mg tablets, one tablet daily for five consecutive days each month.

**FIGURE 2:**
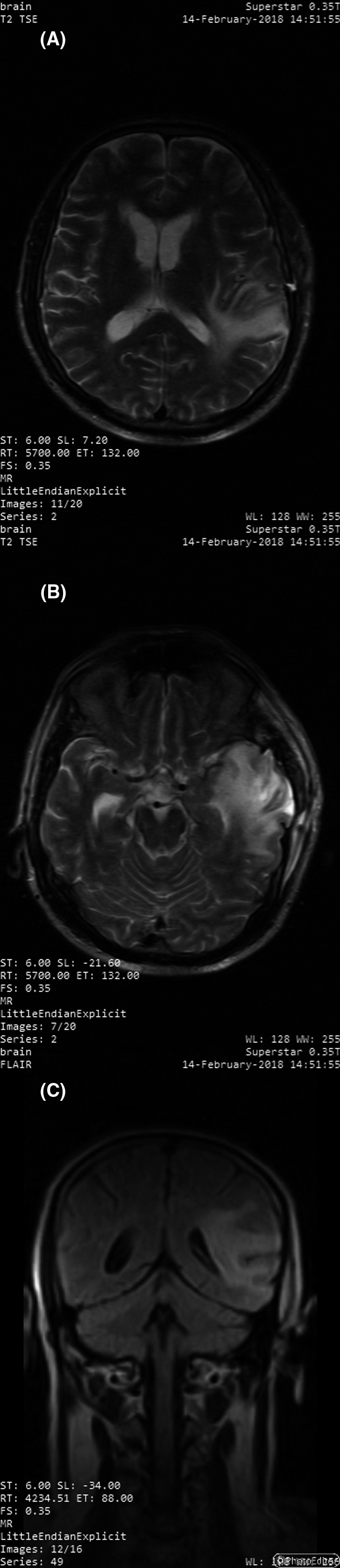
MRI 4 mo after the surgery showing the area of the lesion with residual edema with no midline deviation nor ventricular compression on transverse T2 TSE images (A and B) and coronal FLAIR image (C)

A follow‐up MRI 4 months after the surgery with T1, T2, and FLAIR phases revealed no signs of compression, increased intracranial pressure, ventricular dilation or compression, nor falx cerebri deviation. An area of a mixed signal measuring 25 mm in diameter was present in the left temporal lobe corresponding to an accumulation of fluid in the site of surgery. This area was surrounded by irregularly bordered edema with varied coloration after contrast injection but without any detectable local metastasis (Figure 2).

Six months after the surgery, the patient reported an enlargement in his left cheek. Ultrasound (US) of the area revealed hypoechoic lesions with a partially liquid content inside the parotid gland tissue and in the adjacent tissues (largest of which was 15 × 25 mm). The echo image also detected enlarged left jugular lymph nodes whose structure did not contain fatty hila and was not compatible with an inflammatory origin. The US examination was followed by CT imaging that showed an infiltrating contrast‐enhancing mass in the anterior segment of the left temporal lobe inferior to the location of the previous surgery. This mass measured (31 × 54 mm) and expanded inferiorly outside the cranium and reached the area of the left medial pterygoid plate and the zygomatic process of the maxilla, thus infiltrating the insertion of the masseter muscle. Additionally, other lesions were present; a mass measuring (66 × 51 mm) was infiltrating the left parotid gland and the adjacent mandibular ramus with extension toward the carotid space. Necrotic nodal masses were detected posterior to the sternocleidomastoid muscle, the angle of the mandible, and in the supraclavicular fossa, the largest of which measured 30 mm in diameter.

**FIGURE 3:**
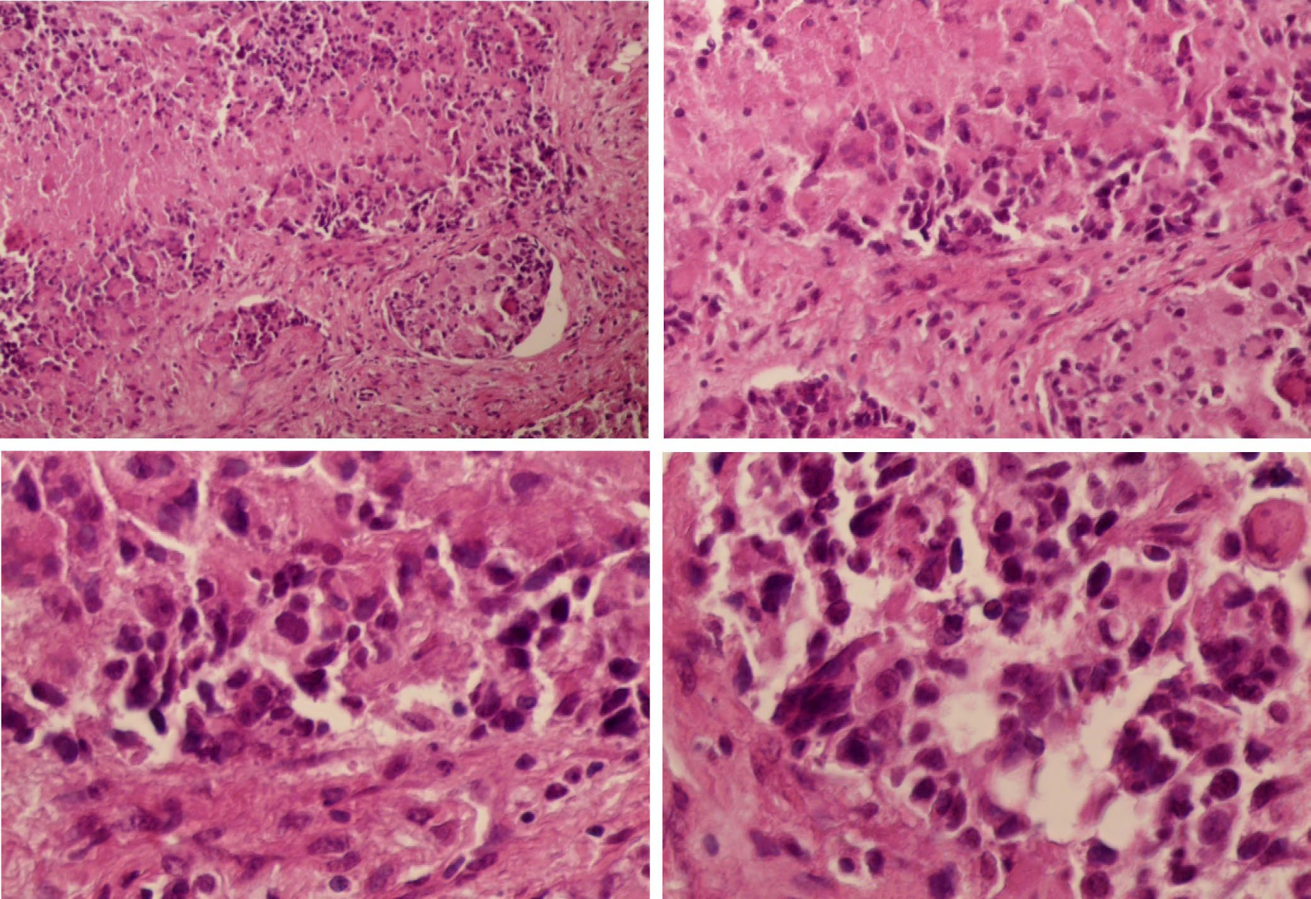
Pathological microscopic images of the lymph node biopsy showing: invasive proliferating anaplastic cells, large hyperchromatic nuclei and occasional bizarre mitotic figures, with no identified residual lymphoid tissue

**FIGURE 4:**
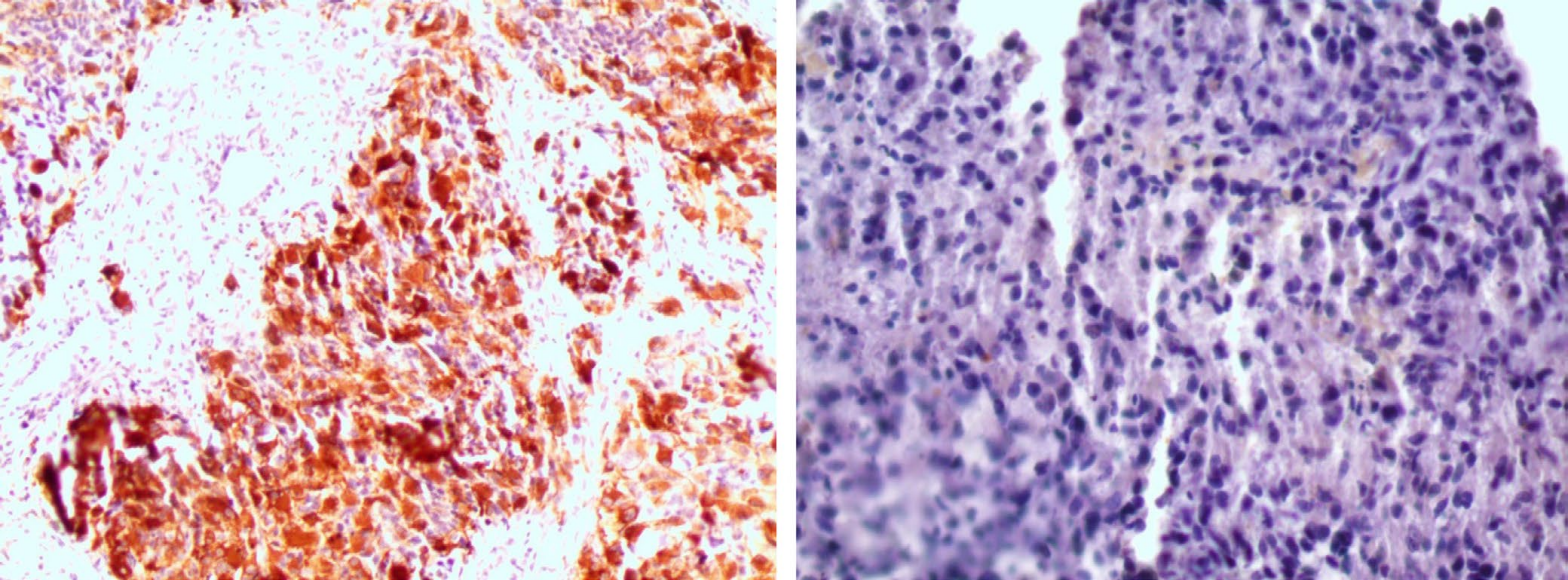
Immune staining of the lymph node biopsy confirming the GBM origin with positive GFAP staining (on the left) and negative CK staining (on the right)

Biopsy of the lymph nodes revealed complete destruction of the lymph node architecture, which was occupied by anaplastic cells. These cells were characterized by large hyperchromatic nuclei, scanty cytoplasm, and occasional bizarre mitotic figures. The surrounding stroma was necrotic and infiltrated by patchy mononuclear inflammatory cell infiltrate (Figure 3). Immune stains were positive for glial fibrillary acidic protein (GFAP) and negative for cytokeratin (CK) (Figure 4). The pathologist concluded that the mass represented a metastasis from glioblastoma multiform. A whole‐body CT scan discovered no other metastases foci in any other organ (including liver, kidney, and spleen).

The patient succumbed to the disease and passed away in September 2018, 4 months after metastasis discovery.

## 3 DISCUSSION

Glioblastoma multiforme is a grade IV diffuse glioma and the most common cerebral tumor in adults.^3^, ^4^ It presents mainly, similar to other high‐grade gliomas, with headache (50%‐60%), focal neurologic symptoms (10%‐40%), and seizures.^11^ It is important to mention that seizures are less likely to occur in glioblastoma in comparison with other lower‐grade gliomas.^11^ Our patient's presentation was typical with severe headache, paraplegia, and slurred speech but no concrete evidence of increased intracranial pressure (no papilledema was seen, but lumbar puncture was not done to rule it out). Further imaging and later surgery, pathology and immune stains confirmed the diagnosis: a contrast‐enhancing lesion with irregular borders on MRI, a necrotic mass of large, pleomorphic, hyperchromatic cells with numerous mitoses, microvascular proliferation, and focal astrocytic differentiation along with positive GFAP staining and negative CK; these findings are consistent with standard GBM diagnostic criteria.^5^ Recent research pointed to the new requirement of testing for isocitrate dehydrogenase (IDH) mutation and 1p/19q codeletion in the process. Based on these mutations, glioblastoma was subclassified into IDH‐wildtype (90% of cases) and IDH‐mutant (10% of cases).^4^ The patient had no known prior neurological malignancy which suggests the IDH‐wildtype as the most favorable; however, testing for these mutations is unavailable in our country and was not performed. Standard treatment involves surgical resection followed by radiation therapy of 60 Gray and temozolomide (75 mg/m^2^ daily up to 49 days) followed by up to six cycles of adjuvant temozolomide (150‐200 mg/m^2^ daily for 5 days, every 28 days),^5^ which was administered accordingly. The efficacy of radiation and temozolomide combination is high and increases survival to 14 months on average.^7^

Metastasis from glioblastoma is rare due to the enclosed environment in which brain tumors grow and their rapid progression before metastases occur. Local implantation was recorded after surgery, while distant metastases were mainly to the spine and less likely to other organs such as lymph nodes, liver, and spleen. The reported cases of metastasis and the mechanism of spreading were vastly investigated in multiple reviews that suggested two possible pathways: The first is the direct invasion through all possible exits (nerves, blood vessels, and meninges and their respective orifices), and the second is the hematological pathway as circulating glioblastoma tumor cells were detected in blood in 20% of the studied cases even before undertaking the surgery.^8^, ^10^, ^12^ The lymphatic pathway has been dismissed for a long time due to the falsely believed absence of lymphatic tissue within the CNS, but recent research was finally able to uncover lymphatic vessels within the meninges and called it “glymphatic system”.^13^ This discovery adds a new possibility on how to interpret the reported cases of distant metastasis, which yet has to be investigated. We found seven cases in literature up‐to‐date where glioblastoma invaded the parotid gland.^14^, ^15^, ^16^, ^17^, ^18^, ^19^, ^20^

In our case, metastasis occurred despite comprehensive excision and the following adjuvant therapy. The etiology of this metastasis could not be established but was most probably preceded by micro‐remnants from the original tumor. These remnants could have multiplied leading to a local recurrence then spread out through cranial base orifices into adjacent tissues. This hypothesis is supported by the diagnostic imaging that revealed a connection between the recurrent tumor and the pterygoid fossa. Later, the tumor could have easily spread from the pterygoid fossa to the masseter and parotid gland. However, micro‐seeding during the surgery is also a logical mechanism to consider, and a lymphatic metastasis could be behind the foci discovered in the lymph nodes. A new surgical intervention by the time of diagnosis was not sought because of the low estimated chances of survival. The patient continued the adjuvant therapy which was not sufficient, and he passed away 9 months after the initial surgical intervention, 4 months after the metastases were diagnosed.

## 4 CONCLUSION

Brain tumors rarely metastasize outside the cranium; however, regional metastasis is a possible event that does not necessarily respond to the current regimens of treatment: surgery, radiation, and temozolomide. Metastases suggested mechanisms are micro‐remnants of the tumor or seeding during the surgery. Lymphatic dissemination is a newly emerging theory that requires further investigation as well. Therefore, special attention should be paid to notice the local invasion and prevent the ominous prognosis in such cases.

## CONFLICT OF INTEREST

We declare no competing interests.

## AUTHOR CONTRIBUTIONS

SA: served as first author and made discussion, abstract, and general reviewing; AA: involved in data collection, literature review, and background; GA: involved in data collection, literature review, and case presentation; MM: contributed to patient's observation and surgery and general reviewing; ZS: served as supervisor and guarantor, and made pathology image reading.

## ETHICS APPROVAL AND CONSENT TO PARTICIPATE

Not required.

## PATIENT CONSENT

Written informed consent was obtained from the patient for publication of this case report and any accompanying images. A copy of the written consent is available for review by the Editor‐in‐Chief of this journal.
